# The Transition of Photoreceptor Guanylate Cyclase Type 1 to the Active State

**DOI:** 10.3390/ijms23074030

**Published:** 2022-04-05

**Authors:** Manisha Kumari Shahu, Fabian Schuhmann, Alexander Scholten, Ilia A. Solov’yov, Karl-Wilhelm Koch

**Affiliations:** 1 Division of Biochemistry, Department of Neuroscience, University of Oldenburg, 26111 Oldenburg, Germany; manisha.kumari.shahu1@uni-oldenburg.de (M.K.S.); alexander.scholten@uni-oldenburg.de (A.S.); 2 Institute of Physics, University of Oldenburg, 26111 Oldenburg, Germany; fabian.schuhmann@uni-oldenburg.de (F.S.); ilia.solovyov@uni-oldenburg.de (I.A.S.); 3 Research Centre for Neurosensory Science, University of Oldenburg, 26111 Oldenburg, Germany

**Keywords:** guanylate cyclase, cGMP, calcium-binding proteins, phototransduction, retinal degeneration, vision, retina, GCAP, molecular-dynamics simulation, protein geometry

## Abstract

Membrane-bound guanylate cyclases (GCs), which synthesize the second messenger guanosine-3′, 5′-cyclic monophosphate, differ in their activation modes to reach the active state. Hormone peptides bind to the extracellular domain in hormone-receptor-type GCs and trigger a conformational change in the intracellular, cytoplasmic part of the enzyme. Sensory GCs that are present in rod and cone photoreceptor cells have intracellular binding sites for regulatory Ca^2+^-sensor proteins, named guanylate-cyclase-activating proteins. A rotation model of activation involving an α-helix rotation was described as a common activation motif among hormone-receptor GCs. We tested whether the photoreceptor GC-E underwent an α-helix rotation when reaching the active state. We experimentally simulated such a transitory switch by integrating alanine residues close to the transmembrane region, and compared the effects of alanine integration with the point mutation V902L in GC-E. The V902L mutation is found in patients suffering from retinal cone–rod dystrophies, and leads to a constitutively active state of GC-E. We analyzed the enzymatic catalytic parameters of wild-type and mutant GC-E. Our data showed no involvement of an α-helix rotation when reaching the active state, indicating a difference in hormone receptor GCs. To characterize the protein conformations that represent the transition to the active state, we investigated the protein dynamics by using a computational approach based on all-atom molecular dynamics simulations. We detected a swinging movement of the dimerization domain in the V902L mutant as the critical conformational switch in the cyclase going from the low to high activity state.

## 1. Introduction

Membrane-bound guanylate cyclases (GCs) are single-pass transmembrane proteins that operate as key enzymes in diverse physiological processes by synthesizing the second messenger guanosine-3′, 5′-cyclic monophosphate (cGMP). The functional state of the enzyme requires a homodimeric topology, and the different GC subgroups are built from a similar molecular domain structure that consists of an extracellular domain (ECD), a transmembrane domain (TM), a kinase homology domain (KHD), a dimerization domain (DD), and a catalytic domain (CCD). GC subgroups, however, differ remarkably in their regulatory features. Extracellular ligands, as natriuretic peptides, activate hormone-receptor GCs and regulate blood pressure, skeletal growth, and water transport. A second subgroup operates in sensory cells that mediate phototransduction, chemosensation, and thermosensation [1,2,3]. GC-E and GC-F (also dubbed ROS-GC1/2 and retGC1/2) are expressed in vertebrate rod and cone photoreceptor cells. These enzymes are not activated by external ligands; they are instead regulated in the cytoplasmic area by guanylate-cyclase-activating proteins (GCAPs) in response to changes in free cytoplasmic [Ca^2+^]. GCAPs operate in a gradual step-by-step activation mode, which shapes the photoreceptor light response [4,5,6,7,8]. Several regions in photoreceptor GC-E participate in binding and/or regulation by GCAPs [9,10,11], but the interaction mode and the location of binding sites in the target GCs are still a matter of discussion [10,11]. Mammalian photoreceptor GCs further bind to several other signaling proteins (for a review, see [12]), including the retinal degeneration protein 3 (RD3), which has a strong inhibitory effect and is involved in GC trafficking [13,14]. The molecular mechanism of these control steps is unknown so far. 

Membrane-bound, hormone-regulated GCs are in a preformed dimeric but an inactive state prior to ligand binding or before receiving a triggering activation signal. A rotation mechanism was proposed for the natriuretic peptide-receptor A (GC-A) involving the juxtamembrane region that is located between the TM and KHD regions (for a review, see [15,16]). This hypothesis of an activating rotation mechanism was experimentally investigated by testing mutants of GC-A for GC activity by successive integration of up to five alanine residues close to the N-terminal end of the TM region. The main conclusion of this work was that binding of the natriuretic peptide ligand triggered an α–helix rotation in the TM domain, which was further transmitted to the CCD [17]. By this mechanism, GC-A switched from the inactive to the active form. However, it is unknown whether all membrane GCs or even all transmembrane cell-surface receptors operate via this mechanism [16]. 

Sensory GCs such as GC-E and GC-F are not activated by external ligands. Switching these GCs to the active state requires a change in free [Ca^2+^] that is detected by guanylate-cyclase-activating proteins (GCAPs) acting as intracellular Ca^2+^ sensor proteins and activity regulators of GC-E and GC-F [2,4,5,6,7,8]. This activation step differs fundamentally from those triggered by the binding of extracellular ligands. Further, the photoreceptor GC-E can interact with mutant GCAP1 variants, forming a constitutively active state under physiological free [Ca^2+^] that persists even under conditions of nonphysiologically high free [Ca^2+^]. These mutations in GCAP1 correlate with forms of retinal diseases, and have been discussed as the molecular cause of visual impairment diagnosed in affected patients [18,19,20,21,22,23,24,25,26]. More recently, Wimberg et al. (2018) described a valine/leucine point mutation in human GC-E in amino acid position 902 (V902L mutant) [27]. This amino acid substitution leads to a constitutively active GC-E showing only a small additional activation by GCAP1 or GCAP2. The point mutation apparently facilitates a conformational change to the active state of GC-E that does not need the stabilizing regulatory interaction with either one or two of the GCAP Ca^2+^ sensors. This conformational transition appears to be similar to the results obtained with the alanine mutants of GC-A, in which the integration of four alanine residues enforced a helix rotation, leading to constitutive GC activation [17]. 

In the present study, we discussed whether the rotation model of activation was a common activation motif among membrane-bound GCs or not. We tested whether we could induce a similar constitutive activation of GC-E by integrating alanine residues close to the TM region. We compared the effects of alanine integration with the point mutation V902L in GC-E, which led to a constitutively active state of GC-E, and correlated with cone–rod dystrophy in a patient [27]. We further analyzed the impact of the point mutation through molecular-dynamics simulations using three-dimensional structural data of bovine GC-E.

## 2. Results

### 2.1. Test of the Rotation Model in the GC-E Activation Process

**Figure 1:**
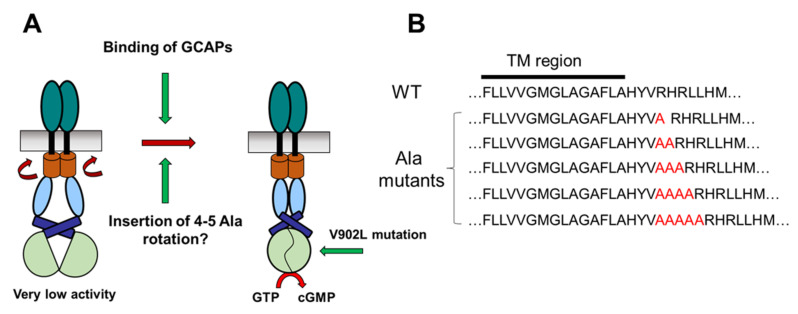
Hypothesis regarding photoreceptor GC-E reaching the active state. (**A**) Activation of GC-E by GCAPs could trigger a rotation in the cytoplasmic part of GC-E, leading to the active state. The point mutation V902L leads to a constitutively active state of human GC-E [27]. (**B**) Insertion of Ala residues (red) after the TM region of human GC-E.

Integration of one alanine residue in an α-helical secondary structural region would induce a helix rotation of 100°. Therefore, a complete 360° rotation could originate from four alanine residues inserted into the primary sequence. If a rotation of the TM region or other domains in the GC-E structure was an essential step for the transition to the active state, we expected a constitutive activity of GC-E in the absence of GCAPs (Figure 1A). Further, we predicted no Ca^2+^ dependency of the GC-E activity. To challenge our hypotheses, we cloned GC-E constructs that harbored up to five additional alanine residues (Figure 1B) and tested the heterologous expression of the recombinant GC-E mutants in HEK293 cells. All GC-E variants were expressed in the HEK293 cells and were suitable for subsequent functional tests. The expression was probed by employing immunoblotting with anti-GC-E antibodies in the HEK293 cell-membrane preparations (Supplementary Figure S1).

**Figure 2:**
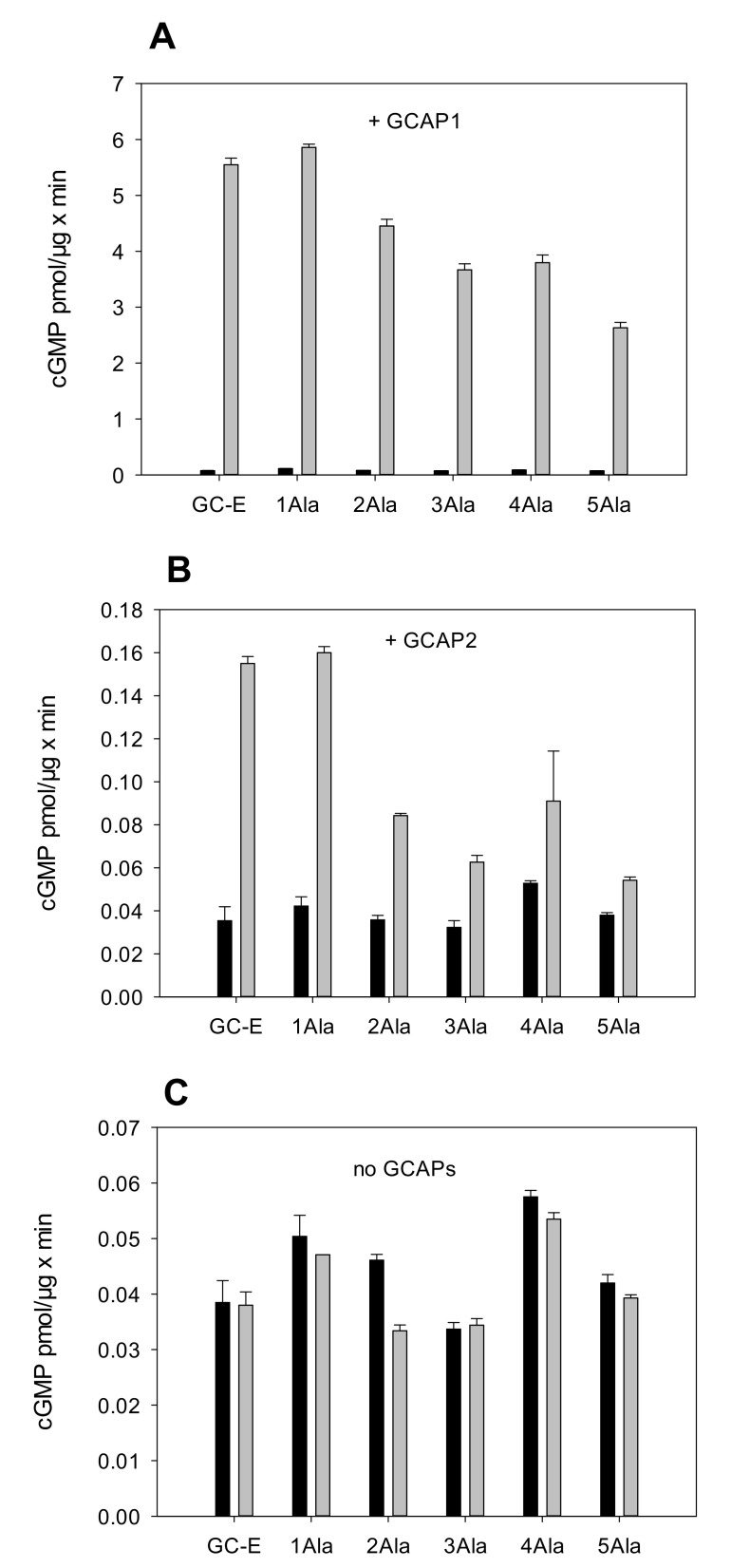
GC activities of Ala mutants and WT human GC-E. (**A**) Ala mutants were incubated at low (<10 nM, grey bars) and high (33 µM, black bars) free [Ca^2+^] in the presence of 5 µM human GCAP1 (*n* = 3). Control incubation of WT GC-E with GCAP1. (**B**) Similar to A, GCAP2 was present instead of GCAP1 (*n* = 3). (**C**) Incubation of Ala mutants and WT GC-E in the absence of GCAPs at high and low [Ca^2+^] (*n* = 3). Results show basal GC activities that indicate no activating effect by the insertion of Ala residues.

Enzymatic GC assays were performed in the presence and absence of GCAP1 and GCAP2 at high and low [Ca^2+^]. GC activities were compared with those obtained with wild-type (WT) GC-E. Figure 2 summarizes the activity profiles of GC-E variants and the regulatory impact of GCAP1 and GCAP2 on the WT and the alanine mutants at low (grey bars) and high (black bars) free [Ca^2+^]. First, the activity of WT GC-E showed a strong dependency on free [Ca^2+^] and GCAP1, but it was less sensitive to GCAP2 (Figure 2A,B; notice the different scaling), which agreed with previous observations [18,19,27]. Integration of more than one alanine residue caused a 30–50% decrease in GCAP1 sensitivity without having a significant effect on the Ca^2+^-inhibited state (Figure 2A). Testing the alanine mutants in the presence of GCAP2 resulted in a similar outcome. A low activation rate of WT GC-E by GCAP2 was observed when the alanine mutants were incubated with GCAP2, yielding either identical (1Ala in Figure 2B) or 50–70% lower activation levels (Figure 2B). Comparing WT and mutant GC activities in the absence of GCAPs (only in the presence of the incubation buffer) showed nearly similar basal GC activities at a very low level (Figure 2C).

In summary, the data in Figure 2 showed that none of the alanine mutants exhibited a constitutive activity of GC-E, indicating that the activation mechanism of GC-E appears to be different from that hypothesized for natriuretic peptide receptor A (GC-A).

### 2.2. Kinetic Analysis of the Constitutively Active GC-E Mutant V902L

**Figure 3:**
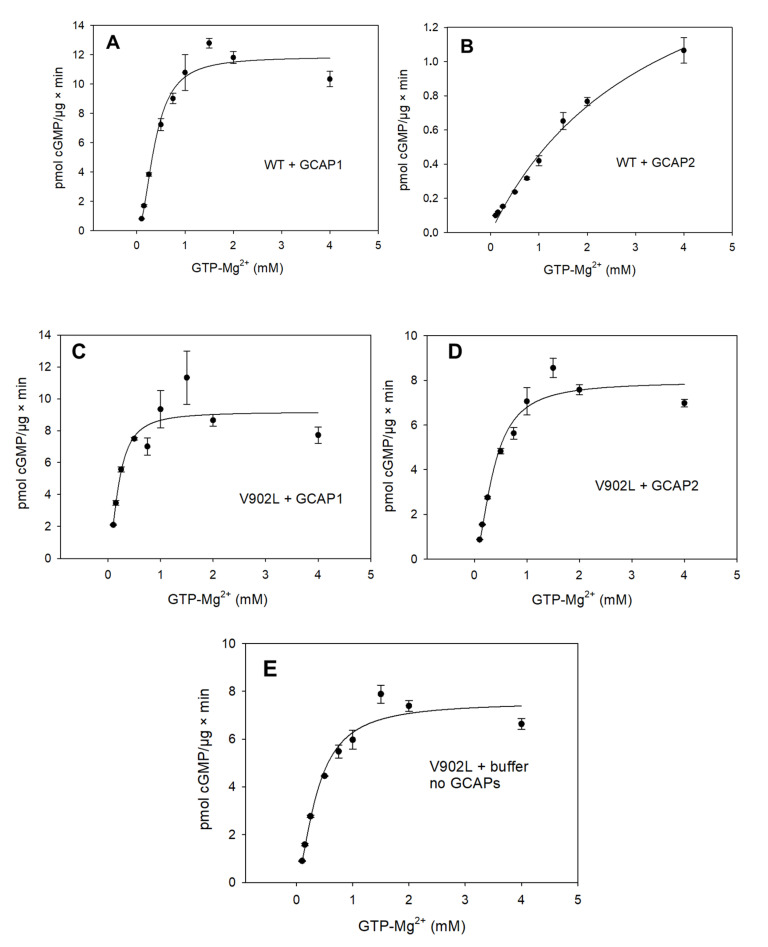
Functional analysis of WT and V902L mutant of human GC-E. Human GC-E variants were expressed in HEK293 cells and incubated with GCAP1 or GCAP2 at increasing Mg-GTP concentrations (in mM). Activities of WT GC-E in the presence of 5 µM GCAP1 (**A**) or 5 µM GCAP2 (**B**) (*n* = 3). (**C**,**D**) The corresponding data sets obtained with the V902L mutant. (**E**) Constitutive activity of V902L in the absence of GCAPs. (*n* = 3).

The point mutation V902L in human GC-E causes cone–rod dystrophy (CRD), and so far is unique among mutations correlating with CRD in patients [27,28]. The mutation transforms GC-E into a constitutively active state that displays only limited GCAP sensitivity. To gain more insight into this active state and the mechanism underlying its transition, we performed a series of enzymatic assays. We tested the GC activity of WT and V902L mutants as a function of the substrate (Mg-GTP) concentration, both in the presence and absence of GCAP1 and GCAP2. Direct plots of GC activity versus (Mg-GTP) showed, in all cases, a sigmoidal curve indicating a cooperative process (Figure 3). Activation rates of GC-E in the presence of GCAP1 became saturated above a substrate concentration of 1 mM GTP, reaching a Vmax of 11.33 pmol/µg × min (Figure 3A). Similar results were obtained for the V902L mutant (Figure 3C,D). The WT GC-E showed a 10-fold lower activation rate due to GCAP2 (Figure 3B; note the different scaling), in agreement with previous reports [18,19,27], and the curve did not reach saturation over the tested concentration range of Mg-GTP. All other activation curves became saturated at Vmax values between 7 and 11 pmol/min/µg of protein. Figure 3E demonstrates the constitutively active state of the V902L mutant in the absence of GCAPs, reaching half-maximal saturation at 0.37 mM GTP (Table 1). Fitting of the curves to a Hill model (see methods) yielded values for Vmax, half-maximal saturation (EC_50_), and the apparent Hill coefficient, as listed in Table 1.

**Figure 4:**
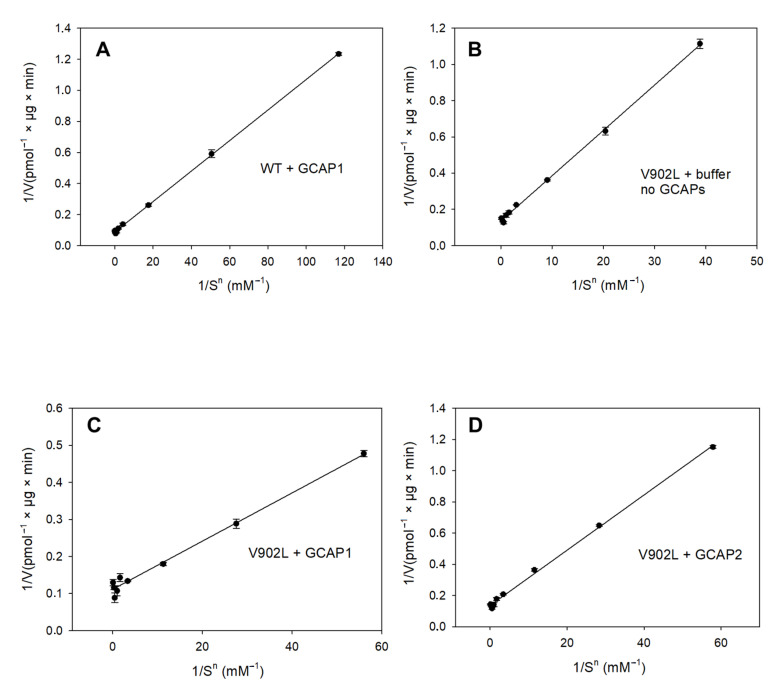
Lineweaver–Burk plot analysis of GC activities. GC-E variants were analyzed as indicated: WT GC-E in the presence of GCAP1 (**A**) and mutant V902L (**B**) in the absence of GCAPs. V902L mutant in the presence of GCAP1 (**C**) and GCAP2 (**D**). Reciprocal substrate GTP concentration 1/S is given in (mM^−1^). Kinetics, as shown in Figure 3, showed deviations from an ideal Michaelis–Menten model. We linearized the plots by using apparent Hill coefficients of h = 2.07 (WT + GCAP1), h = 1.58 (V902L + buffer, no GCAPs), h = 1.75 (V902L + GCAP1), and h = 1.76 (V902L + GCAP2).

We tested the results for Vmax and EC_50_ (equivalent to an apparent K_M_) through analysis of Lineweaver–Burk plots. Due to the cooperativity of substrate binding, we could weigh the *x*-axis 1/[S] with a corresponding Hill coefficient n (1/[S]^n^) to yield straight lines (Figure 4). The analysis revealed apparent K_M_ values for the WT and the V902L mutant, which were nearly identical to the determination of the corresponding EC_50_ values (Table 1). We limited the analysis to WT GC-E + GCAP1 and the mutant V902L + GCAP1/GCAP2, because human WT GC-E showed very low activation rates due to GCAP2 (Figure 3B; also see [27]), and we reached no saturation with the substrate in a reasonable concentration range. An analysis of the results shown in Figure 3B, however, revealed a half-maximal saturation (EC_50_) at 3.45 mM GTP, which was at least 10-fold higher than the K_M_ determined for the combination of WT GC-E with GCAP1, V902L with GCAP1 or GCAP2, and V902L without GCAPs (Table 1). This result confirmed the much lower activation rate of human WT GC-E by GCAP2, which could originate from a 10-fold lower affinity of human WT GC-E for the Mg-GTP substrate.

The analysis showed further that the constitutively active state of the V902L mutant did not differ significantly from the active state of the WT GC-E with respect to the catalytic parameters Vmax, K_M_, and k_cat_, yielding similar catalytic efficiencies between 2000 and 4000 M^−1^ × s^−1^ (expressed in k_cat_/K_M_; Table 1, Figure 3 and Figure 4).

We concluded from these results that the active state of the V902L mutant originated from a protein conformation that was similar to the conformation of the WT GC-E in the active state stabilized by GCAP1 interaction. To gain more insight into the protein conformations that represented the transition to the active state, we undertook a computational approach based on all-atom molecular-dynamics (MD) simulations.

### 2.3. Molecular-Dynamics Simulations

**Figure 5:**
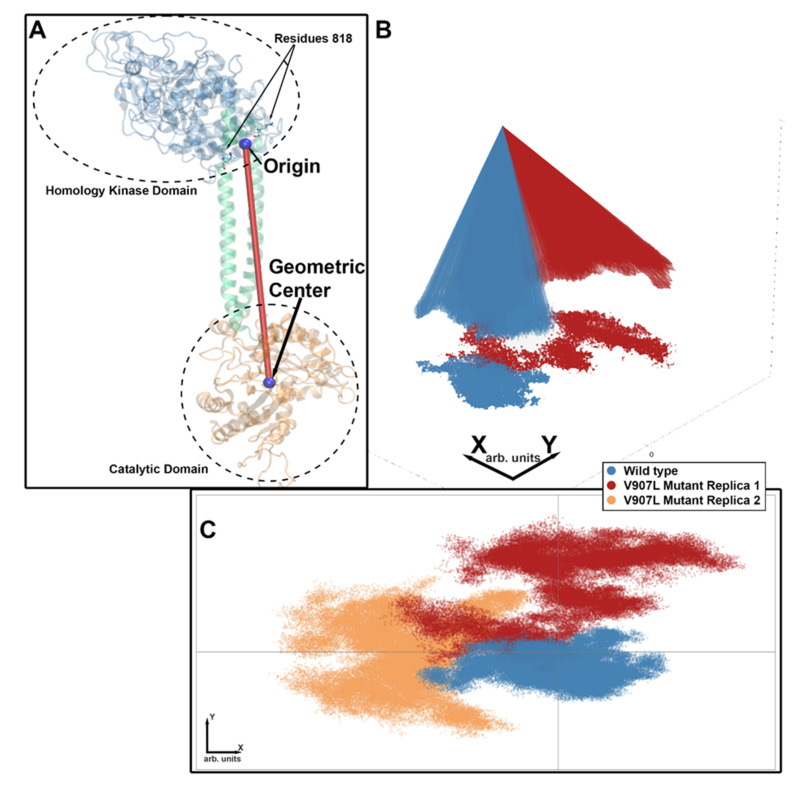
Visualization of bovine GC-E orientation through the plane projection. (**A**) The two residues at position 818 in the GC-E dimer were located at the beginning of the alpha-helical dimerization domain (green). The protein structure was translated such that the origin (0,0,0) was just in the middle between the two residues 818 (measured from the first carbon in the backbone). For each MD snapshot, the line segment between the origin and the geometric center of the catalytic domain was considered as indicated by the red line in panel A. (**B)** The endpoints of the line segments were then projected to the x–y plane. The projection describes the area in which the catalytic domain moved relative to the kinase homology domain during the simulation. (**C**) The last panel shows the top view of the projected area, including the results for both mutant replica simulations. It can be seen that the wild type remained in its original position, while both replicas moved away, although in different directions.

**Figure 6:**
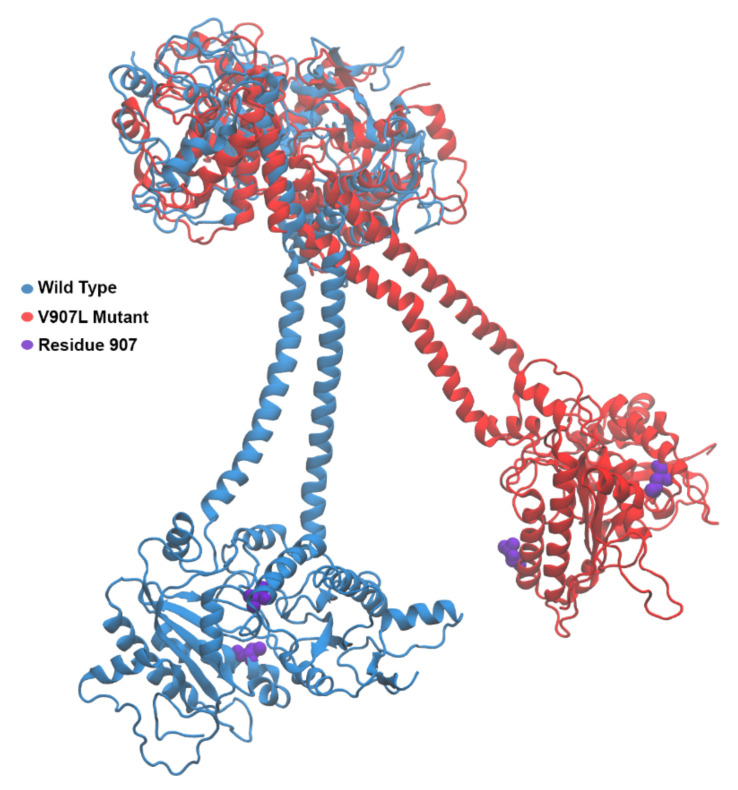
Bovine GC-E protein structure. Starting from the top, the kinase homology domain is shown, which is followed by the alpha-helical dimerization domain, and concludes in the catalytic domain. The wild-type structure is shown in blue, and the V907L mutant is shown in red. Occurrences of residue 907 are shown in purple.

The amino acid residue at position 902 is in the catalytic domain, and the mutation might cause a significant reorganization of the dimeric arrangement in this domain. Simulations were based on the structural information recently reported by Rehkamp et al. [29] for bovine GC-E. Human and bovine orthologues share a high sequence identity/homology, and the corresponding valine is in position 907 (mutant V907L in the MD approach; amino acid numbering of the modeled structure refers to the bovine orthologue). Previous work identified the GCAP1 binding sites in the juxtamembrane and kinase homology region of mammalian GC-E [10,11,30,31], indicating a movement or conformational change of the catalytic domain relative to the kinase homology domain during the activation process. To probe for the catalytic domain movement, the kinase homology domain of the WT bovine GC-E had to be aligned to the structure of the mutant (see Section 4 for details). The photoreceptor GC-E exists as a homodimer [32]. The two residues in position 818 are located just before the alpha-helical dimerization domain. The residues were taken as a reference point, and the structure was translated such that the middle between the two residues 818 coincided with the origin of the coordinate system used for analysis (see Figure 5). For each MD snapshot, the geometric center of the catalytic domain was calculated and used to define the catalytic domain’s position. The line segment connecting the origin to the geometric center described the tilting of the alpha-helical and catalytic domains relative to the kinase homology domain. To show the changes arising during bovine GC-E dynamics, the endpoints of the line segments were projected onto a plane, as visualized in Figure 5 and Figure 6.

### 2.4. Difference Distance Matrix

**Figure 7:**
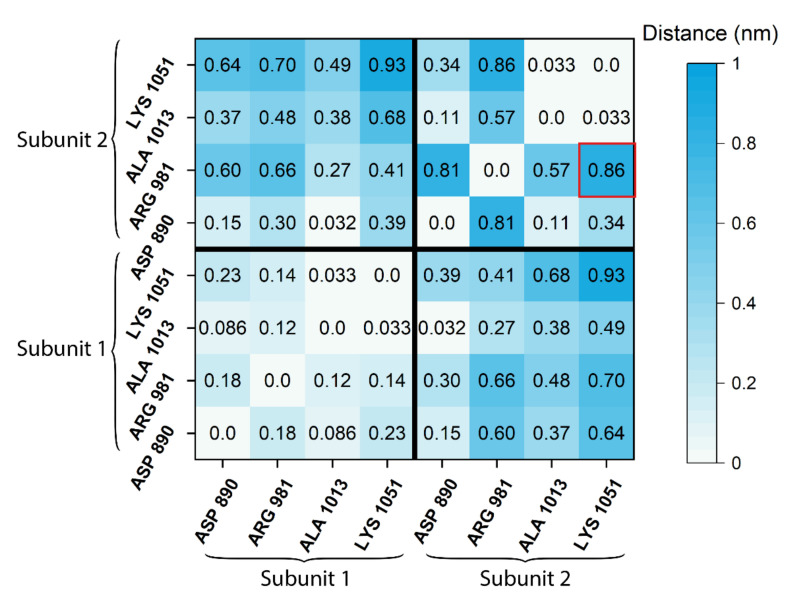
Difference distance matrix for binding-site residues. The eight residues comprising the binding site are shown in a symmetric difference distance matrix, which quantifies the change in distance throughout the simulation duration between the wild type and the V907L mutant of the bovine GC-E. The first four residues belong to the first subunit, while the last four belong to the second. The lower-left and upper-right submatrices compare distances within a subunit, while the bottom right shows the differences beyond the individual subunits. For instance, the highlighted entry (red square) indicates that the average change in distance from Lys1051 to Arg981 from the second subunit is 0.86 nm when comparing the wild type to the mutant.

The GTP substrate-binding site in photoreceptor GC-E is thought to comprise residues Asp890, Arg981, Ala1013, and Lys1051 in the cyclase catalytic domain [33,34]. The alignment process was repeated while aligning the whole structure, and hence not excluding the catalytic domain. Difference distance matrices were computed following the approach outlined earlier [35], in which every residue was described through the first backbone carbon atom, and the distances to all other seven binding-site residues were measured. These calculated distances were averaged over the duration of the MD simulation. The process was repeated for the WT and the V907L mutant of GC-E. This analysis resulted in two 8 × 8 matrices containing the pairwise averaged distances with zero diagonal elements. The element-wise difference of the two matrices yielded a difference distance matrix that showed the positional changes of the residue relative to the other trajectory (Figure 7), which made the conformational change measurable.

**Figure 8:**
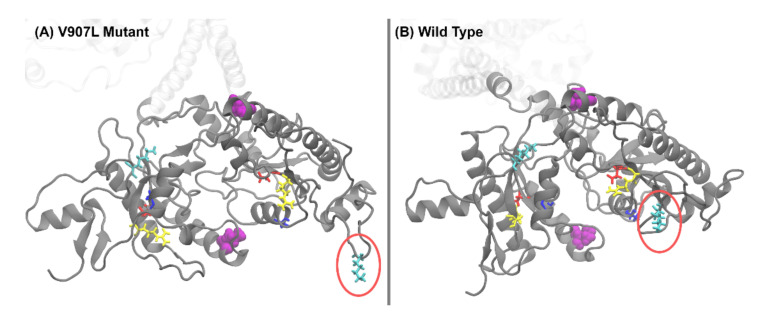
Critical amino acid positions in the putative GTP substrate-binding site in GC-E. Panels (**A**) and (**B**) show the catalytic domain for the V907L mutant and WT GC-E, respectively. The residues comprising the binding site are represented by sticks and colored according to their type (blue = Ala, lime = Arg, red = Asp, cyan = Lys). In one of the subunits of the GC-E structure, Lys1051 exhibits significant movement as it swings out (red ellipsoid). Occurrences of residue 907 are highlighted in purple.

Data in the difference distance matrix indicated that the residues in one subunit moved very little relative to each other after the mutation, while the other subunit experienced profound changes. In particular, Lys1051 changed its position drastically (visually shown in Figure 8). Furthermore, the distance between the two sets of four residues changed noticeably. It came as a surprise that the point mutation V907L had asymmetric effects on the structure of the catalytic domain.

## 3. Discussion

Membrane-bound GCs reach the active state by different interaction processes. Hormone-receptor GC types bind hormone peptides at the extracellular domain, whereas photoreceptor GCs have intracellular binding sites for Ca^2+^-sensor proteins, named GCAPs. However, despite these apparent differences, membrane-bound GCs could reach the active state by similar conformational transitions, since membrane-bound GCs share a similar structural topology. Experimental support for a ligand-induced rotation mechanism exists for GC-A [17]. We used the same experimental approach as Parat et al. [17] by inserting alanine residues close to the TM region (Figure 1), and tested the functional consequences. Since we did not observe any constitutive activation of GC-E, we interpreted our results as showing that the successive insertion of alanine residues did not cause a conformational transition to the active state. In fact, the alanine point mutants displayed some minor disturbances in basal and GCAP-dependent activity, pointing to a mechanism that did not involve a rotational repositioning of the TM or juxtamembrane region.

These results left an unanswered question regarding the conformational dynamics exhibited by photoreceptor GCs in their transit to the active state. We took advantage of the point mutation V902L in human GC-E, which was characterized as permanently active without the on–off control operated by the GCAP Ca^2+^ sensor. So far, the V902L mutation in GC-E is the only point mutation known so far in human GC-E that leads to a constitutive activation in the absence of GCAPs. Other point mutations in GC-E causing visual impairment showed drastically reduced activities or a change in Ca^2+^-sensitive GCAP regulation [28], and not an increase in basal enzyme activity. Thus, we used this mutant as a tool to investigate the mechanism of GC-E activation. Previous work on V902L focused on the effects of changing Ca^2+^ and GCAP concentrations, but not on the substrate dependency and the catalytic efficiency expressed in k_cat_/K_M_. We determined these values as listed in Table 1, showing that k_cat_/K_M_ for the mutant V902L with GCAP1 and without GCAPs was similar to the parameter analyzed for the WT with GCAP1. The constitutive activity of the V902L mutant could not, therefore, originate from a higher catalytic efficiency. In fact, rod and cone photoreceptor GCs are low-efficiency enzymes with k_cat_/K_M_ of 1.9 × 10^4^–1.6 × 10^5^ M^−1^s^−1^, as noted previously for the bovine and mice orthologues [5,6].

The parameter k_cat_/K_M_ was equivalent to the association rate constant of the substrate binding to the enzyme. Taking structural information as visualized for the bovine orthologue in Figure 8, the V907L mutation caused a flexible movement of a Lys in position 1051, swinging away from the dimer interface. This movement or higher flexibility might facilitate reaching the transition state in the catalytic center. GCAPs most likely activate photoreceptor GCs by stabilizing the transition state [36]. In the constitutively active state of GC-E, the stabilization must be realized by the point mutation and its structural consequences. Figure 6 illustrates how the swinging movement of the alpha-helical domain connected to the catalytic domain leads to a different orientation of the residues in position 907. In the WT GC-E, both valines were close to the dimer interface and opposite to each other. In the mutant, Leu907 was more exposed on the protein surface. Leu contains one additional methylene group in the side chain. The side chain is longer and more hydrophobic than in Val. This might have caused a repulsion between the leucines, leading to a different dimer interface.

Constitutive activation of human GC-E is a consequence of several point mutations that cause retinal dystrophies in patients suffering from visual impairment [28]. Most of these mutations are, however, found in the regulatory Ca^2+^ sensor GCAP1. Point mutations in GCAP1 very often cause lower affinities for Ca^2+^, and GCAP1 mutants thereby display a shift in Ca^2+^-sensitive GC regulation to free [Ca^2+^], which is outside of the physiologically relevant range between 50 and 500 nM that leads to permanent activity of the GCAP1/GC-E complex [18,19,20,21,22,23,24,25,26]. Retinal-disease-related point mutations in the alpha-helical dimerization domain of human GC-E also correlated in some cases with a disturbance of Ca^2+^-sensitive control by GCAPs [9] (for a review, see [28]). The alpha-helical dimerization domain has been discussed as a Ca^2+^-sensitive control module in this context that is influenced by Ca^2+^-dependent conformational changes in GCAPs, but is not a Ca^2+^ sensor itself [9]. The swinging movement of the dimerization domain in the V902L mutant, as illustrated in Figure 6, might represent the critical conformational switch in the cyclase going from the low to high activity state. In the case of the WT, the switch was triggered by interaction with GCAP1; in the case of the V902L mutant, the amino acid exchange was sufficient for the transition. Our results showed a principal difference in the activation modus between hormone receptor GCs and sensory GCs, which is relevant to the understanding of the molecular basis of retinal diseases.

## 4. Materials and Methods

### 4.1. Cloning of Polyalanine GC-E Mutants

To create the five desired polyalanine GC-E mutants, the WT GC-E sequence was cloned into a pIRES2-eGFP vector and used as a template [9]. Site-directed mutagenesis was achieved by introducing one to five alanines at the C-terminal site of the transmembrane region (Figure 1B) in the GC-E sequence by polymerase chain reaction (PCR) using a KOD (Hot start DNA Polymerase Novagen^®^) enzyme. Instructions according to the manufacturer’s protocol were followed. The primers used to produce the mutants are listed in Supplementary Table S1. The GC-E polyalanine mutants are abbreviated as **1Ala**, **2Ala**, **3Ala**, **4Ala**, and **5Ala**. Since the cloning strategy failed when creating 4Ala, we used a different approach. The isolated 1Ala cDNA was used instead of GC-E cDNA to introduce four alanine residues at the site of interest while employing the primer set *b,* as mentioned in Supplementary Table S1. The obtained clones were verified by full-length sequencing of the GC-E coding region. The vector encoding the V902L mutant sequence [27] was retransformed into XL-1 Blue cells for DNA isolation and for further expressing the mutant protein transiently in HEK 293 cells.

### 4.2. Heterologous Expression of GC-E, Polyalanine Mutants, and V902L Mutant

For further functional studies, the HEK293 cell line (Thermo Fisher Scientific, Germany) was transiently transfected with GC-E WT cDNA and the respective mutant forms. Transfection was performed using polyethylenimine (PEI) at 60–70% of confluency in 100 mm plates. A total of 8 µg of DNA was mixed with 32 µg of PEI in DMEM without supplements and incubated at room temperature (RT) for 15 min. Subsequently, the sample mix was added to the respective cell plates and incubated in the incubator at 37 °C, 5% CO_2_. Cells were harvested for 72–96 h of incubation after transfection by centrifugation for 5 min at 500× *g*. Cell pellets were washed with PBS, transferred into 1.5 mL tubes, and centrifuged again for 5 min at 12,000× *g*. The pellets were frozen at −80 °C until further use.

### 4.3. Electrophoresis and Western Blotting

Sodium dodecyl–sulfate polyacrylamide gel electrophoresis (SDS-PAGE) and Western blotting were performed according to established procedures in the laboratory [9,37] with the following modification: the protein transfer was done via a tank-transfer method. The blot was then incubated in TBST buffer (Tris-buffered saline/0.05% Tween 20) with 1X ROTI^®^Block (ROTH) solution for 1 h. The primary antibody GC1 #3 directed against bovine GC-E [5,9] recognized the human GC-E, and was used at a dilution of 1:10,000. Incubation with the primary antibody was done overnight at 4 °C. A goat antirabbit peroxidase-conjugated antibody (Dianova, Germany) at a concentration of 50% in glycerol was used as a secondary antibody at a dilution of 1:5000.

### 4.4. Guanylate Cyclase Assay and Enzyme Kinetics

To analyze GC-E activity for a possible effect of subunit rotation, the enzymatic activity of the polyalanine mutants compared to WT GC-E was measured. Transfected HEK cell pellets were resuspended in 1 mL of 10 mM Hepes/KOH pH 7.4 with 1 mM DTT and a protease inhibitor cocktail. The suspension was incubated on ice for 30 min followed by cell lysis using a syringe with a 0.7 mm needle. After centrifugation at 13,000× *g* for 8 min at 4 °C, the cell pellet was resuspended in 100 µL of 50 mM Hepes/KOH pH 7.4, 50 mM KCl, 20 mM NaCl, 1 mM DTT, and a protease inhibitor cocktail. A total of 20 µL of a GCAP1 or GCAP2 solution (5 µM) or water, which were previously adjusted to different free Ca^2+^ concentrations using a Ca^2+^/EGTA buffer system, were used exactly as described previously [5,9,38]. For each sample, 10 µL of respective membrane suspensions were mixed and preincubated for 5 min at room temperature. The reaction was started by adding 20 µL of 2.5 × GC buffer (75 mM Mops/KOH pH 7.2, 150 mM KCl, 10 mM NaCl, 2.5 mM DTT, 8.75 mM MgCl_2_, 2.5 mM GTP, 0.75 mM, and 0.4 mM Zaprinast). The reaction mixtures were incubated for 10 min at 30 °C and stopped by adding 50 µL 0.1 M EDTA and incubating at 95 °C for 5 min. The samples were centrifuged for 10 min at 13,000× *g*. The supernatants were analyzed for produced cGMP by reversed-phase HPLC using a LiChrospher^®^ 100 RP-18 (5 μm) column (Merck, Darmstadt, Germany) exactly as described previously [5,9,38].

For the analysis of enzyme kinetics of the WT GC-E and the mutant V902L, we basically followed our previous protocol [5]. Briefly, GC-E variants were reconstituted with 5 µM of purified myristoylated forms of human GCAP1 and human GCAP2. Incubations containing V902L were also done in the absence of GCAPs. The guanylate cyclase activity was assayed at 2 mM EGTA (≤10 nM free Ca^2+^) as a function of the substrate GTP (concentration range between 0 and 4 mM). The analysis of data was performed as previously reported [5]. Plots of activity versus Mg-GTP concentration were fitted to a Hill model (f = a × x^h^/(c^h^ + x^h^)) using SigmaPlot 13.0.

### 4.5. Expression and Purification of GCAP1 and GCAP2

Human myristoylated GCAP1 and GCAP2 were expressed in *E. coli* and purified to homogeneity by anion-exchange chromatography and size-exclusion chromatography as previously reported [5,38]. Myristoylation of GCAPs during bacterial expression was accomplished by cotransforming *E. coli* cells with N-myristoyl-transferase from yeast and supplementation with myristic acid, as reported previously [5]. We modified the expression of GCAP1 for obtaining the protein in a soluble fraction using 0.1 mM IPTG at 30 °C for 2 h.

### 4.6. Expression of the Catalytic Domain of GC

A fusion construct consisting of the catalytic domain of GC-E and the maltose-binding protein (MBP) was expressed and purified as previously reported [11], but with the addition of a further purification step using a Ni-NTA column. A fraction containing the GC-E construct was loaded on a Ni-NTA column that was pre-equilibrated with 20 mM Tris/HCl pH 7.5, 150 mM NaCl, 5% (*v/v*) glycerol, 10 mM imidazole, and 5 mM β-mercaptoethanol. After loading, the column was washed with the same buffer before the construct was eluted using 20 mM Tris/HCl pH 7.5, 200 mM NaCl, 5% glycerol, 250 mM imidazole, and 5 mM β-mercaptoethanol. The eluted protein fractions were stored in aliquots at −80 °C until further use.

### 4.7. Chemiluminescence Detection and Quantification of Polyalanine Mutants

Expression of polyalanine mutants: 5 µg of protein of each variant was analyzed by SDS-PAGE and Western blotting as described above. After transfer to the blot membrane, blots were incubated with Western Bright ECL reagent 1 and 2 (advansta), and protein bands were detected using the Azure c400 Gel Imaging System by Azure Biosystems. The intensity of protein bands was determined using the device-specific software AzureSpot (Supplementary Figure S1). The difference in the protein expression level of mutant proteins was normalized to the WT GC-E. The normalization of varying amounts in protein expression level was considered while calculating the final cGMP level.

### 4.8. Quantification of WT GC-E and V902L Mutant

The fusion construct MBP-CD, consisting of the catalytic domain of GC-E and MBP, was used for creating a calibration curve. For this purpose, increasing amounts of purified MBP-CD (20–400 ng) were applied on SDS polyacrylamide gels. Membrane suspensions of 2.5 µg and 5 µg of total membrane protein containing GC-E or V902L were applied on the same gels and analyzed by using SDS-PAGE. After electrophoresis, the proteins were blotted, and the band intensity was detected and determined as described above using Azure c400 and AzureSpot. Varying amounts of MBP-CD were used for calibration, and the amounts of cyclase GC-E and V902L were obtained from the calibration curve (Supplementary Figure S2).

### 4.9. Molecular Dynamics Simulation

In a recent study [29] the bovine guanylate cyclase 1 protein from the rod outer segment membrane bovine GC-E was modeled. The resulting published structure serves as a base for the following all-atom molecular dynamics MD protocol.

The studied structure contains the kinase homology domain, the alpha-helical domain, and the catalytic domain, while the extracellular, transmembrane domain and juxtamembrane domain are missing. Additionally, residues 608 to 617 are missing in the structure, but the sequence for these residues is known. The structure of bovine GC-E was used to study the behavior of its human counterpart, as it is in close resemblance with a sequence identity of 87% in the existing parts. Due to a slight shift in sequence comparing the bovine to the human GC-E protein, all residues are off by five. Hence, assuming one is looking for residue 902 in human GC-E, one would choose residue 907 in bovine GC-E. All simulations were carried out using the bovine GC-E structure.

All simulations were run using NAMD [39,40], and the interatomic interactions were described using the CHARMM36 force field with CMAP corrections [41,42,43,44,45,46,47,48]. Simulations were set up using the online platform VIKING [49].

The peptide modeler Pep McConst [35] was used to reconstruct the missing residues, followed by structure minimization in a vacuum, in which all residues except the newly added ones were restrained to allow the newly added part to relax to a feasible conformation. Following energy minimization, the structure was first equilibrated with a simulation timestep of 0.25 fs, which was increased to 1.0 fs once a stable conformation was achieved. The equilibrated structure was solvated in a water box and neutralized. A total of 4 mM NaCl and 3.5 mM MgCl_2_ were added to neutralize and ionize the system. The system was then equilibrated further and subsequently simulated extensively at a temperature of 310 K and an atmospheric pressure of 1 bar in an NVT (constant number of particles, volume, and temperature) ensemble. In total, the structure was equilibrated for over 140 ns.

The alignment was conducted by an iterative scheme employing the Kabsch algorithm successfully applied in an earlier study [50]. The simulated trajectory was aligned to the first snapshot as a reference, and then iteratively aligned to the new average structure until the overall root-mean-square deviation dropped below a threshold value.

Two replica simulations in which the valine residue at position 907 in bovine GC-E was mutated to leucine (V902L) were initiated. The wild-type simulation was also continued. The mutations were conducted using the Mutator plugin of VMD [51]. The three simulations (WT and two replicas of V907L) were conducted for 400 ns each. The resulting trajectories were prepared for analyses using the MDTraj package [52].

## Figures and Tables

**Table 1:** Catalytic parameters of WT GC-E and V902L.

Sample	Vmax (pmol/µg × min)	EC_50_ (mM)	K_M_ (mM)	Kcat (s^−1^)	Kcat/K_M_ (10^3^ M^−1^ × s^−1^)	Hill Parameter h
WT GC-E + GCAP1	11.33	0.37	0.34	0.8	2.35	2.07
WT GC-E + GCAP2	-	3.45	-	-	-	-
V902L + GCAP1	9	0.20	0.20	0.8	4	1.75
V902L + GCAP2	7.32	0.36	0.31	0.7	2.2	1.76
V902L no GCAPs	7.36	0.37	0.34	0.7	2.1	1.58

## Data Availability

Data are stored on institutional (university-operated) devices; they are available upon request from the corresponding authors.

## Supplementary Materials

The following supporting information can be downloaded at: https://www.mdpi.com/article/10.3390/ijms23074030/s1.

## References

[1] Pichlo M., Bungert-Plümke S., Weyand I., Seifert R., Bönigk W., Strünker T., Kashikar N.D., Goodwin N., Müller A., Pelzer P. (2014). High density and ligand affinity confer ultrasensitive signal detection by a guanylyl cyclase chemoreceptor. J. Cell Biol..

[2] Sharma R.K., Duda T., Makino C.L. (2016). Integrative Signaling Networks of Membrane Guanylate Cyclases: Biochemistry and Physiology. Front. Mol. Neurosci..

[3] Kuhn M. (2016). Molecular Physiology of Membrane Guanylyl Cyclase Receptors. Physiol. Rev..

[4] Mendez A., Burns M.E., Sokal I., Dizhoor A.M., Baehr W., Palczewski K., Baylor D.A., Chen J. (2001). Role of guanylate cyclase-activating proteins (GCAPs) in setting the flash sensitivity of rod photoreceptors. Proc. Natl. Acad. Sci. USA.

[5] Hwang J.Y., Lange C., Helten A., Höppner-Heitmann D., Duda T., Sharma R.K., Koch K.W. (2003). Regulatory modes of rod outer segment membrane guanylate cyclase differ in catalytic efficiency and Ca(2+)-sensitivity. Eur. J. Biochem..

[6] Peshenko I.V., Olshevskaya E.V., Savchenko A.B., Karan S., Palczewski K., Baehr W., Dizhoor A.M. (2011). Enzymatic properties and regulation of the native isozymes of retinal membrane guanylyl cyclase (RetGC) from mouse photoreceptors. Biochemistry.

[7] Makino C.L., Wen X.H., Olshevskaya E.V., Peshenko I.V., Savchenko A.B., Dizhoor A.M. (2012). Enzymatic relay mechanism stimulates cyclic GMP synthesis in ro; photoresponse: Biochemical and physiological study in guanylyl cyclase activating protein 1 knockout mice. PLoS ONE.

[8] Koch K.W., Dell’Orco D. (2013). A calcium-relay mechanism in vertebrate phototransduction. ACS Chem. Neurosci..

[9] Zägel P., Dell’Orco D., Koch K.W. (2013). The dimerization domain in outer segment guanylate cyclase is a Ca^2^⁺-sensitive control switch module. Biochemistry.

[10] Peshenko I.V., Olshevskaya E.V., Dizhoor A.M. (2015). Evaluating the role of retinal membrane guanylyl cyclase 1 (RetGC1) domains in binding guanylyl cyclase-activating proteins (GCAPs). J. Biol. Chem..

[11] Sulmann S., Kussrow A., Bornhop D.J., Koch K.W. (2017). Label-free quantification of calcium-sensor targeting to photoreceptor guanylate cyclase and rhodopsin kinase by backscattering interferometry. Sci. Rep..

[12] Koch K.W., Dell’Orco D. (2015). Protein and Signaling Networks in Vertebrate Photoreceptor Cells. Front. Mol. Neurosci..

[13] Azadi S., Molday L.L., Molday R.S. (2010). RD3, the protein associated with Leber congenital amaurosis type 12, is required for guanylate cyclase trafficking in photoreceptor cells. Proc. Natl. Acad. Sci. USA.

[14] Peshenko I.V., Olshevskaya E.V., Azadi S., Molday L.L., Molday R.S., Dizhoor A.M. (2011). Retinal degeneration 3 (RD3) protein inhibits catalytic activity of retinal membrane guanylyl cyclase (RetGC) and its stimulation by activating proteins. Biochemistry.

[15] Misono K.S., Philo J.S., Arakawa T., Ogata C.M., Qiu Y., Ogawa H., Young H.S. (2011). Structure, signaling mechanism and regulation of the natriuretic peptide receptor guanylate cyclase. FEBS J..

[16] Maruyama I.N. (2015). Activation of transmembrane cell-surface receptors via a common mechanism? The “rotation model”. Bioessays.

[17] Parat M., Blanchet J., De Léan A. (2010). Role of juxtamembrane and transmembrane domains in the mechanism of natriuretic peptide receptor A activation. Biochemistry.

[18] Dell’Orco D., Dal Cortivo G. (2019). Normal GCAPs partly compensate for altered cGMP signaling in retinal dystrophies associated with mutations in GUCA1A. Sci. Rep..

[19] Avesani A., Marino V., Zanzoni S., Koch K.W., Dell’Orco D. (2021). Molecular properties of human guanylate cyclase-activating protein 2 (GCAP2) and its retinal dystrophy-associated variant G157R. J. Biol. Chem..

[20] Kitiratschky V.B., Behnen P., Kellner U., Heckenlively J.R., Zrenner E., Jagle H., Kohl S., Wissinger B., Koch K.W. (2009). Mutations in the GUCA1A gene involved in hereditary cone dystrophies impair calcium-mediated regulation of guanylate cyclase. Hum. Mutat..

[21] Dizhoor A.M., Boikov S.G., Olshevskaya E.V. (1998). Constitutive activation of photoreceptor guanylate cyclase by Y99C mutant of GCAP-1. Possible role in causing human autosomal dominant cone degeneration. J. Biol. Chem..

[22] Sokal I., Li N., Surgucheva I., Warren M.J., Payne A.M., Bhattacharya S.S., Baehr W., Palczewski K. (1998). GCAP1 (Y99C) mutant is constitutively active in autosomal dominant cone dystrophy. Mol. Cell.

[23] Biasi A., Marino V., Dal Cortivo G., Maltese P.E., Modarelli A.M., Bertelli M., Colombo L., Dell’Orco D. (2021). A Novel GUCA1A Variant Associated with Cone Dystrophy Alters cGMP Signaling in Photoreceptors by Strongly Interacting with and Hyperactivating Retinal Guanylate Cyclase. Int. J. Mol. Sci..

[24] Marino V., Dal Cortivo G., Oppici E., Maltese P.E., D’Esposito F., Manara E., Ziccardi L., Falsini B., Magli A., Bertelli M. (2018). A novel p.(Glu111Val) missense mutation in GUCA1A associated with cone-rod dystrophy leads to impaired calcium sensing and perturbed second messenger homeostasis in photoreceptors. Hum. Mol. Genet..

[25] Vocke F., Weisschuh N., Marino V., Malfatti S., Jacobson S.G., Reiff C.M., Dell’Orco D., Koch K.W. (2017). Dysfunction of cGMP signalling in photoreceptors by a macular dystrophy-related mutation in the calcium sensor GCAP1. Hum. Mol. Genet..

[26] Peshenko I.V., Cideciyan A.V., Sumaroka A., Olshevskaya E.V., Scholten A., Abbas S., Koch K.W., Jacobson S.G., Dizhoor A.M. (2019). A G86R mutation in the calcium-sensor protein GCAP1 alters regulation of retinal guanylyl cyclase and causes dominant cone-rod degeneration. J. Biol. Chem..

[27] Wimberg H., Lev D., Yosovich K., Namburi P., Banin E., Sharon D., Koch K.W. (2018). Photoreceptor Guanylate Cyclase (GUCY2D) Mutations Cause Retinal Dystrophies by Severe Malfunction of Ca^2+^-Dependent Cyclic GMP Synthesis. Front. Mol. Neurosci..

[28] Sharon D., Wimberg H., Kinarty Y., Koch K.W. (2018). Genotype-functional-phenotype correlations in photoreceptor guanylate cyclase (GC-E) encoded by GUCY2D. Prog. Retin. Eye Res..

[29] Rehkamp A., Tänzler D., Tüting C., Kastritis P.L., Iacobucci C., Ihling C.H., Kipping C., Koch K.-W., Sinz A. (2021). First 3D-Structural Data of Full-Length Guanylyl Cyclase 1 in Rod-Outer-Segment Preparations of Bovine Retina by Cross-Linking/Mass Spectrometry. J. Mol. Biol..

[30] Lange C., Duda T., Beyermann M., Sharma R.K., Koch K.W. (1999). Regions in vertebrate photoreceptor guanylyl cyclase ROS-GC1 involved in Ca(2+)-dependent regulation by guanylyl cyclase-activating protein GCAP-1. FEBS Lett..

[31] Krylov D.M., Hurley J.B. (2001). Identification of proximate regions in a complex of retinal guanylyl cyclase 1 and guanylyl cyclase-activating protein-1 by a novel mass spectrometry-based method. J. Biol. Chem..

[32] Yang R.B., Garbers D.L. (1997). Two eye guanylyl cyclases are expressed in the same photoreceptor cells and form homomers in preference to heteromers. J. Biol. Chem..

[33] Tucker C.L., Hurley J.H., Miller T.R., Hurley J.B. (1998). Two amino acid substitutions convert a guanylyl cyclase, RetGC-1, into an adenylyl cyclase. Proc. Natl. Acad. Sci. USA.

[34] Liu Y., Ruoho A.E., Rao V.D., Hurley J.H. (1997). Catalytic mechanism of the adenylyl and guanylyl cyclases: Modeling and mutational analysis. Proc. Natl. Acad. Sci. USA.

[35] Schuhmann F., Korol V., Solov’yov I.A. (2021). Introducing Pep McConst—A user-friendly peptide modeler for biophysical applications. J. Comput. Chem..

[36] Koch K.W. (2002). Target recognition of guanylate cyclase by guanylate cyclase-activating proteins. Adv. Exp. Med. Biol..

[37] Scholten A., Koch K.W. (2011). Differential calcium signaling by cone specific guanylate cyclase-activating proteins from the zebrafish retina. PLoS ONE.

[38] Koch K.W., Helten A. (2008). Guanylate cyclase-based signaling in photoreceptors and retina. Signal Transduction in the Retina.

[39] Phillips J.C., Braun R., Wang W., Gumbart J., Tajkhorshid E., Villa E., Chipot C., Skeel R.D., Kalé L., Schulten K. (2005). Scalable Molecular Dynamics with NAMD. J. Comput. Chem..

[40] Phillips J.C., Hardy D.J., Maia J.D., Stone J.E., Ribeiro J.V., Bernardi R.C., Buch R., Fiorin G., Hénin J., Jiang W. (2020). Scalable Molecular Dynamics on CPU and GPU Architectures with NAMD. J. Chem. Phys..

[41] Foloppe N., MacKerell A.D. (2000). All-Atom Empirical Force Field for Nucleic Acids: I. Parameter Optimization Based on Small Molecule and Condensed Phase Macromolecular Target Data. J. Comput. Chem..

[42] Best R.B., Zhu X., Shim J., Lopes P.E.M., Mittal J., Feig M., MacKerell A.D. (2012). Optimization of the Additive CHARMM All-Atom Protein Force Field Targeting Improved Sampling of the Backbone ϕ, ψ and Side-Chain χ1 and χ2 Dihedral Angles. J. Chem. Theory Comput..

[43] Hart K., Foloppe N., Baker C.M., Denning E.J., Nilsson L., MacKerell A.D. (2012). Optimization of the CHARMM Additive Force Field for DNA: Improved Treatment of the BI/BII Conformational Equilibrium. J. Chem. Theory Comput..

[44] Pavelites J.J., Gao J., Bash P.A. (1997). A Molecular Mechanics Force Field for NAD^+^, NADH, and the Pyrophosphate Groups of Nucleotides. J. Comput. Chem..

[45] MacKerell A.D., Banavali N.K. (2000). All-Atom Empirical Force Field for Nucleic Acids: II. Application to Molecular Dynamics Simulations of DNA and RNA in Solution. J. Comput. Chem..

[46] Denning E.J., Priyakumar U.D., Nilsson L., MacKerell A.D. (2011). Impact of 20-Hydroxyl Sampling on the Conformational Properties of RNA: Update of the CHARMM All-Atom Additive Force Field for RNA. J. Comput. Chem..

[47] MacKerell A.D., Feig M., Brooks C.L. (2004). Improved Treatment of the Protein Backbone in Empirical Force Fields. J. Am. Chem. Soc..

[48] MacKerell A.D., Bashford D., Bellott M., Dunbrack R.L., Evanseck J.D., Field M.J., Fischer S., Gao J., Guo H., Ha S. (1998). All-atom Empirical Potential for Molecular Modeling and Dynamics Studies of Proteins. J. Phys. Chem. B.

[49] Korol V., Husen P., Sjulstok E., Nielsen C., Friis I., Frederiksen A., Salo A.B., Solov’yov I.A. (2019). Introducing VIKING: A Novel Online Platform for Multiscale Modeling. ACS Omega.

[50] Schuhmann F., Kattnig D.R., Solov’yov I.A. (2021). Exploring Post-activation Conformational Changes in Pigeon Cryptochrome 4. J. Phys. Chem. B.

[51] Humphrey W., Dalke A., Schulten K. (1996). VMD: Visual Molecular Dynamics. J. Mol. Graph..

[52] McGibbon R.T., Beauchamp K.A., Harrigan M.P., Klein C., Hernández C.X., Schwantes C.R., Wang L.P., Lane L.P., Pande V.S. (2015). MDTraj: A Modern Open Library for the Analysis of Molecular Dynamics Trajectories. Biophys. J..

