# Supplementary material for: The Transition of Photoreceptor Guanylate Cyclase Type 1 to the Active State
Source: Int J Mol Sci. 2022 Apr 5;23(7):4030. doi: 10.3390/ijms23074030 (PMC8999790; doi:10.3390/ijms23074030)
Supplement: Supplementary file 1 [file ijms-23-04030-s001.zip › ijms-1629703-supplementary.pdf]

Supplementary information

The transition of photoreceptor guanylate cyclase type 1 to the active state

Manisha Kumari Shahu[1], Fabian Schuhmann[2], Alexander Scholten[1], Ilia A. Solv'yov[2,3], Karl-Wilhelm Koch[1,3#]

[1]Division of Biochemistry, Department of Neuroscience, University of Oldenburg, Oldenburg 26111, Germany.

[2]Institute of Physics, University of Oldenburg, Oldenburg 26111, Germany.

[3]Research Centre for Neurosensory Science, University of Oldenburg, Oldenburg 26111, Germany.

*To whom correspondence should be addressed: karl.w.koch@uni-oldenburg.de

Table S1. Oligonucleotide sequences used in the present study.

Mutants	Oligonucleotide Sequence 5'-3'
1 Alanine (1Ala)	**FP-** 5'- AGG CAC CGG CTA CTT CAC ATG CAA ATG -3'<br><br>**RP-** 5'- **AGC** CAC ATA ATG GGC CAG GAA GGC -3'
2 Alanine (2Ala)	**FP-** 5'- AGG CAC CGG CTA CTT CAC ATG CAA ATG -3'<br><br>**RP** 5'- **TGC AGC** CAC ATA ATG GGC CAG GAA GGC -3'
3 Alanine (3Ala)	**FP** 5'- **GCC** AGG CAC CGG CTA CTT CAC ATG -3'<br><br>**RP** 5'- **TGC AGC** CAC ATA ATG GGC CAG GAA GGC -3'
4 Alanine (4Ala) Set a	**FP** 5'- **GCC GCT** AGG CAC CGG CTA CTT CAC ATG -3'<br><br>**RP** 5'- **TGC AGC** CAC ATA ATG GGC CAG GAA GGC -3'
4 Alanine (4Ala) Set b	**FP** 5'- **GCC GCT GCT** AGG CAC CGG CTA CTT CAC ATG -3'<br><br>**RP-** 5'- **AGC** CAC ATA ATG GGC CAG GAA GGC -3'
5 Alanine (5Ala)	**FP** 5'- **GCC GCT GCT** AGG CAC CGG CTA CTT CAC ATG -3'<br><br>**RP** 5'- **TGC AGC** CAC ATA ATG GGC CAG GAA GGC -3'

Figure S1

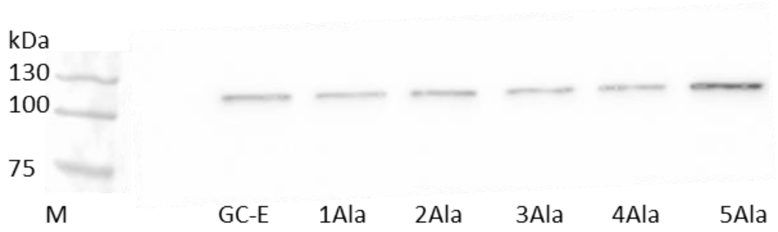

Figure S1. Western blot for verification and quantification of poly alanine mutants. Expression level of mutants was compared to that of GC-E wildtype in HEK293 cell membranes. Five µg of total protein amount for each sample was analysed by SDS-PAGE. The samples were detected by the specific antibody anti-GC1#3. The slight difference between each mutant band intensity was considered for the activity measurement of amount of cGMP synthesized with each sample.

Figure S2

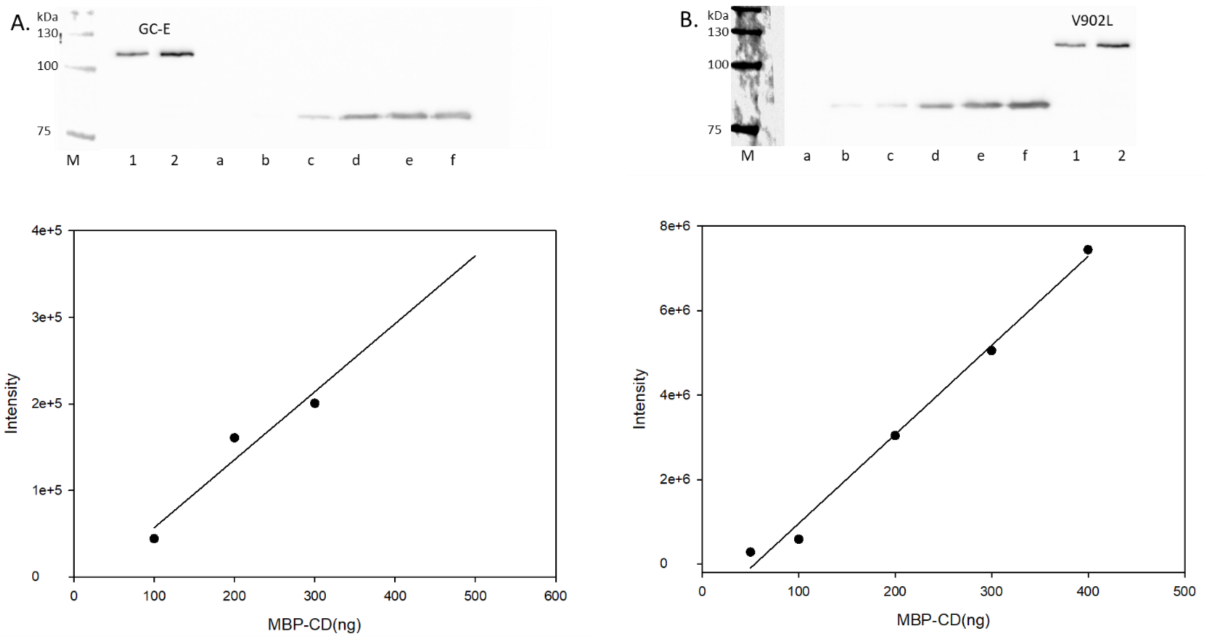

Figure S2. Quantification of GC-E and V902L in HEK293 cell membranes. Purified MBP-CD protein samples were electrophoresed and blotted in increasing amount (a- 20ng, b-50ng, c-100ng, d-200ng, e-300ng, f-400ng) were used to create a calibration curve along with a sample of wildtype GC-E (**A**) or V902L (**B**) present in HEK293 membrane fractions (1- 2.5 µg, 2- 5 µg). The samples were detected by GC1 # 3 antibody and ECL imaging. Band intensity was linear within the tested range for **B**, for **A** the plot was extended manually based on linearity. Quantification yielded for 5 µg membrane protein 292 ng of GC-E wildtype, and 212 ng of V902L, taking a purity of 73% into account.
